# How Should Plant Resistance to Herbivores Be Measured?

**DOI:** 10.3389/fpls.2017.00663

**Published:** 2017-04-26

**Authors:** Johan A. Stenberg, Anne Muola

**Affiliations:** ^1^Department of Plant Protection Biology, Swedish University of Agricultural SciencesAlnarp, Sweden; ^2^Department of Ecology, Swedish University of Agricultural SciencesUppsala, Sweden; ^3^Environmental and Marine Biology, Åbo Akademi UniversityTurku, Finland

**Keywords:** antibiosis, antixenosis, herbivory, integrated pest management, nonpreference, plant breeding, plant defense, resistance breeding

## Introduction

Plant resistance is normally defined as the heritable ability of plants to escape attacking enemies, partially or fully, thus minimizing the amount of damage experienced by the plant (Painter, 1951; Mitchell et al., 2016). Plant resistance is pivotal in preventing crop yield loss to herbivores, and, thus, it is important to breed for (Hill et al., 2012). As many national and intergovernmental bodies have firmly endorsed Integrated Pest Management as the new paradigm for plant protection, the importance of resistant varieties is becoming even more important. However, measuring resistance is seldom straightforward, and many different approaches are being used, thus affecting biological interpretations. Choosing an appropriate measure for plant resistance is essential for engineering future varieties for improved plant production security, with less dependence on chemical pesticides. Here we suggest that the method selected to measure resistance should depend on the longevity of the crop (or culture) and the generation time of the herbivore.

## Two different approaches to measure resistance

Plant resistance can broadly be classified as (1) *antixenosis* (or *nonpreference*), i.e., how much damage is suffered or how many herbivore individuals a plant attracts during a specific time period, or as (2) *antibiosis*, i.e., how suitable a plant is for the herbivore (Box 1; Painter, 1951; Kogan and Ortman, 1978). Antibiosis can, in turn, be measured in terms of intrinsic plant traits (e.g., constitutive and/or induced chemical resistance compounds, or physical traits) or in terms of the fitness effects that these resistance traits have on the herbivore (performance or fitness). In his original resistance framework, Painter (1951) also included *tolerance*, i.e., the ability of a plant to withstand herbivory without any decline in yield. Tolerance, however, was later removed from the resistance concept, and allocated its own category (Mitchell et al., 2016), and will not be considered further in this paper. Of the two remaining approaches to measure plant resistance, *antixenosis* is immediately plant focused, considering the extent to which the plant will be able to escape herbivores. From the herbivore's perspective, however, a resistant host plant is a plant that reduces the fitness of the herbivore. *Antibiosis*, then, can seemingly be viewed as a more herbivore-centered view of plant resistance, considering the quality of the plant as food for the herbivore. As we will see, however, both of these paths ultimately become plant focused and can be equally useful measures of plant resistance, albeit for different time scales.

Box 1 **Two principal approaches to measuring plant resistance to herbivores.**

1. Antibiosis (how suitable the plant is for the herbivore)
  - Herbivore fitness or performance (e.g., fertility rate or larval development time)
  - Intrinsic plant traits (chemical, physical) underlying herbivore fitness.
2. Antixenosis (how much damage or how many herbivores a plant attracts)
  - Herbivore presence (number of eggs, larvae, or adults)
  - Herbivore damage (e.g., percentage leaf area removed).

## Pros and cons of the two approaches

### Antixenosis

The ability of plants to avoid colonization by herbivores involves a number of complex interactions with the herbivore. Host-plant acceptance can be disrupted during host finding (over long or short distances), at the time of physical contact, or during probing (Knolhoff and Heckel, 2014). However, from an applied point of view it may not always be crucial to investigate the mechanisms behind antixenosis—what matters is whether the plant is utilized by the herbivore or not. Plant utilization can be investigated in controlled “cafeteria” settings in the laboratory (e.g., Stenberg et al., 2006), but is more commonly examined in common gardens where more (and larger) plants can be tested simultaneously (e.g., Robinson et al., 2012). Measuring antixenosis in common gardens is typically a relatively time-effective and cheap method. However, this approach may also be less robust. If the percentage leaf area removed by herbivores is assessed by eye, a certain degree of subjectivity is inevitable. This uncertainty can be reduced by photographing all leaves and analyzing the damaged area using image analysis software (Abràmoff et al., 2004), but such an approach is typically time consuming, and less feasible in cases when hundreds of experimental plants are included. If, in contrast, the number of herbivore individuals on the plant is counted, rather than damage, this opens up the risk that well-hidden herbivores are missed.

**Figure 1:**
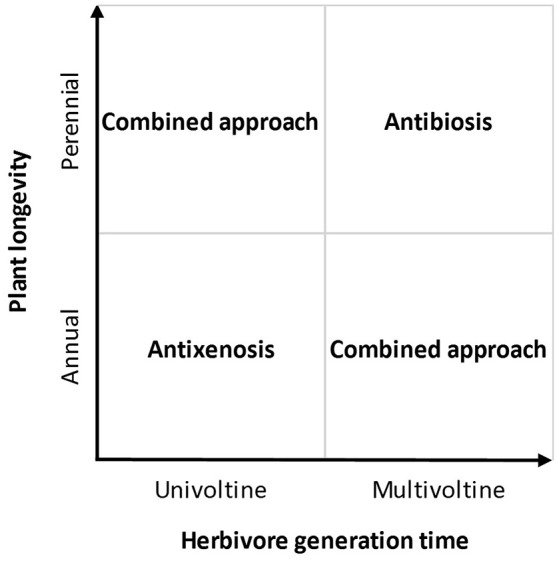
**Schematic showing which approach for measuring plant resistance would be most appropriate depending on the longevity of the crop plant (or culture) and the generation time of the herbivore**.

The most important drawback associated with measures of antixenosis, however, is that damage or herbivore numbers are often only measured during a short period of time, typically using young plants, rarely following the plants over several consecutive years. Thus, the recorded herbivore presence or damage typically reflects behavioral decisions made by colonizing herbivores, ignoring the effects of herbivore population build-up on the different plants. While it is often the case that the parent (colonizing) herbivores select host plants that are palatable for their offspring, this is not always true (Hufnagel et al., 2016). If the lifetime of the plant (or culture) is longer than the generation time of the herbivore, antibiosis will become increasingly important as time passes, while the initial choice of the colonizing herbivores will gradually become less significant (Figure 1).

### Antibiosis

Measuring antibiosis is almost always undertaken in laboratory environments where important factors such as temperature, humidity, and light can be controlled. The dependent variables measured (typically herbivore fertility rate or larval development time in no-choice situations) are often discrete, and relatively unaffected by human subjectivity (e.g., Lehrman et al., 2012). Measuring antibiosis therefore provides the researcher with precise scores of high reliability. If some of the underlying plant traits (e.g., constitutive and/or induced chemical resistance compounds or physical traits) are known, these can be scored as a proxy for antibiosis, if such an approach is more reliable or cost-effective.

Applied researchers have traditionally focused antibiosis on constitutive resistance, forgetting the role of induced resistance (but see Stout, 2013; Mitchell et al., 2016 for modernized concepts that integrate induced and constitutive defenses). A major disadvantage with measuring antibiosis (be it herbivore fitness or underlying plant traits) is that large investments in time and/or equipment are often necessary. Measuring larval development time for herbivores that may take several weeks to develop, or undertaking chemical analyses, is inevitably more demanding than assessing antixenosis in a common garden. For plant breeders, it may often not be feasible to measure antibiosis related parameters on several hundred plant offspring in a crossing population; this would require cheaper standardized methods.

Although antibiosis may intuitively seem less interesting for researchers who are more focused on plant fitness or crop yield, there can be strong arguments for taking this approach. In all situations where the herbivore has the opportunity to establish and remain on a plant or in a culture, the quality of the host plant will ultimately become very important for the population dynamics of the herbivore, and thus for the long-term herbivory experienced by the plant (Ågren et al., 2012).

## Proposed guidelines

Antixenosis and antibiosis are often correlated, because many adult herbivores choose to colonize plants that are palatable to their offspring (e.g., Stenberg et al., 2006). In such cases, it is both scientifically sound and cost-effective to assess antixenosis only. However, there is plenty of evidence showing that simple correlations between antixenosis and antibiosis do not exist for many agriculturally important herbivores (e.g., Hufnagel et al., 2016). For example, generalist herbivores may not always be able to make optimal host plant choices (Gripenberg et al., 2010). In cases when antixenosis or antibiosis are not correlated or their relationship is uncertain, researchers have to choose between the two approaches or combine them.

We suggest that measuring antixenosis is always the most rational option for situations when colonization is more important than the local population dynamics of the herbivore. For example, this is the case when crop plantations are regularly treated with insecticides, which removes the colonizing herbivores before they are able to produce subsequent generations. For low-pesticide cropping systems, however, herbivores often have the opportunity to remain and reproduce. In fact, the whole idea behind integrated pest management is not to eradicate the pests, but to manage them, thus, to some degree, tolerating herbivores within a plantation (Hokkanen, 2015). For those situations, we expect the relative longevity of the herbivore and the crop plant to be of key importance for which approach to choose (Figure 1). In cases when the plant, or crop plantation, is short-lived, and the herbivore has a long generation time (e.g., it is univoltine), then herbivory patterns will mainly depend on the host choices made by the colonizing herbivore individuals, making antibiosis less important. This will often be the case for vegetables that are attacked by univoltine lepidopterans or coleopterans. In contrast, the opposite should be true for fast-growing herbivores such as aphids, mites, and thrips attacking more long-lived crops and orchards. The more herbivore generations that are allowed develop in the crop, the more important antibiosis will be for the long-term population size of the herbivore, and the damage experienced by the plant.

While our model (Figure 1) provides clear and simple directions for two out of four possible situations, it also highlights two more complicated situations where a combined approach including both antixenosis and antibiosis is needed. In such cases, it may be worthwhile to carry out pre-studies investigating whether antixenosis and antibiosis are correlated, as this would allow exclusion of one of them.

## Conclusion

Although measuring resistance is costly and often requires assiduous work, it is likely to provide returns in terms of high, predictable yield with less input of harmful chemical products. However, failing to choose the most suitable measure of resistance may give rise to incorrect conclusions, risking serious pest problems in the field. If, for example, only the antixenosis approach is used to choose “resistant” varieties for a long-lived orchard, this may lead to rapid population build-up of few colonizing herbivores, eventually leading to herbivore outbreak and severe yield losses. If, on the other hand, only the antibiosis approach is used to choose “resistant” varieties for a short-lived vegetable, this may lead to rapid colonization and destruction by adult immigrant herbivores, whose long-term population development is of little importance with respect to the yield. It is our hope that the suggested model (Figure 1) will aid plant breeders and agricultural researchers when choosing approaches to measure resistance that are robust and appropriate for their specific cropping system. If this hope is realized, it will increase the credibility and significance of crop resistance to herbivores and contribute to environmentally-friendly and sustainable agriculture.

## Author contributions

All authors listed, have made substantial, direct and intellectual contribution to the work, and approved it for publication.

## Funding

The authors are funded by The Swedish Research Council Formas (grant nos. 217 - 2014-541 and 216 - 00223), The Crafoord Foundation (grant nos. 20150904 and 20161057), and Carl Tryggers Stiftelse (grant no. CTS 15:468).

### Conflict of interest statement

The authors declare that the research was conducted in the absence of any commercial or financial relationships that could be construed as a potential conflict of interest.
